# Are online prediction tools a valid alternative to genomic profiling in the context of systemic treatment of ER-positive breast cancer?

**DOI:** 10.1186/s11658-017-0049-x

**Published:** 2017-09-04

**Authors:** Umar Wazir, Kinan Mokbel, Amtul Carmichael, Kefah Mokbel

**Affiliations:** The London Breast Institute, Princess Grace Hospital, 45 Nottingham Place, London, W1U 5NY UK

**Keywords:** Breast cancer, Molecular biotechnology, Genomic assay, Prognosis, Gene expression regulation

## Abstract

**Background:**

Clinicians use clinical and pathological parameters, such as tumour size, grade and nodal status, to make decisions on adjuvant treatments for breast cancer. However, therapeutic decisions based on these features tend to vary due to their subjectivity. Computational and mathematical algorithms were developed using clinical outcome data from breast cancer registries, such as Adjuvant! Online and NHS PREDICT. More recently, assessments of molecular profiles have been applied in the development of better prognostic tools.

**Methods:**

Based on the available literature on online registry-based tools and genomic assays, we evaluated whether these online tools could be valid and accurate alternatives to genomic and molecular profiling of the individual breast tumour in aiding therapeutic decisions, particularly in patients with early ER-positive breast cancer.

**Results and conclusions:**

Early breast cancer is currently considered a systemic disease and a complex ecosystem with behaviour determined by the complex genetic and molecular signatures of the tumour cells, mammary stem cells, microenvironment and host immune system. We anticipate that molecular profiling will continue to evolve, expanding beyond the primary tumour to include the tumour microenvironment, cancer stem cells and host immune system. This should further refine therapeutic decisions and optimise clinical outcome.

This article was specially invited by the editors and represents work by leading researchers.

## Introduction

Traditionally, clinicians use clinical and pathological parameters, such as tumour size, grade, nodal status, HER2, ER status and proliferation index, to make decisions regarding adjuvant treatments for breast cancer. However, therapeutic decisions based on these features tend to vary due to their subjectivity [1, 2].

The modern approach uses computational and mathematical algorithms that were developed using clinical outcome data from cancer registries. Adjuvant! Online and NHS PREDICT are examples of such decision-supporting tools. They are freely available online, making them attractive in resource-constrained healthcare settings. They help clinicians to assess an individual’s risk of developing recurrent disease and/or dying within 10 years, and have the potential to guide decisions regarding adjuvant and neoadjuvant therapy [3, 4].

The deepening understanding of breast cancer has been used to significantly improve these types of prognostic tool. A particularly influential discovery was the characterisation of breast cancer as a heterogeneous group of neoplastic processes arising from the ductal or lobular epithelium rather than a single disease with a variable ER and HER2 expression. It enabled the development of better prognostic tools based on assessing molecular profiles. Examples of such assays include Blueprint [5] Mammaprint [6], Oncotype DX [7], prediction analysis of microarray 50 (PAM50) [8, 9] and EndoPredict (EP) [10]. The EndoPredict Clinical (EpClin) assay is a composite of standard pathological parameters and molecular profiling scores which has been found to provide superior prognostic information [11]. These assays have changed the landscape of clinical oncology and allowed clinicians to make therapeutic decisions based on the molecular machinery of the tumour and data derived from randomised controlled trials.

These commercially available molecular scores have not only been found to be cost-effective, they have become less expensive over the past few years. Despite this, their cost may be an issue in resource-poor settings. An analysis by Reed et al. suggests that the initial outlay on genomic assays are offset by future gains in quality-adjusted patient years [12]. However, cost remains a significant consideration.

In this article, we shall discuss the literature on the online tools mentioned above with a view to evaluating whether they could be valid and accurate alternatives to genomic and molecular profiling of the individual breast tumour in aiding therapeutic decisions in the era of personalized precision medicine, particularly in patients with early ER-positive breast cancer.

### Online prediction tools

The online tools referred to earlier primarily use clinicopathological variables and cancer registry data as the basis of risk prediction. The clinical pathological variables used include age, tumour size and grade, mode of detection, number of lymph nodes involved, ER status, HER2 status, Ki67 status and type of chemotherapy [13]. The strengths and weaknesses of these tools draw from the design and limitations of the registry data on which they are based.

#### Adjuvant! Online

The baseline risk estimation for Adjuvant! Online was derived from the SEER (surveillance, epidemiology and end results) database program, which is a collation of nine databases covering 14% of the US population [14]. The SEER database specifically excludes patients under the age of 35 and over the age of 59 [15] and has limited information on the socio-economic status of subject. There have been concerns regarding the quality of the data about cause of death [16, 17].

The database does not include information regarding the benefits of adjuvant trastuzumab, thereby reducing the utility of Adjuvant! Online in clinical decisions about HER2-positive disease treatment [16, 17]. This deficiency of Adjuvant! Online with regards to HER2-positive disease has significant implications for the prediction of metastatic spread. In a recent in vitro study using murine models, the HER2 status of cells predicted the response to progesterone-induced signalling, with HER2-deficient cells being more likely to migrate and HER2-enriched cells tending towards increased proliferation [18]. This recent evidence underlines the importance of HER2 in predicting prognosis and highlights the significance of this inherent shortcoming in online cancer registry-based prognostic tools.

Adjuvant! Online tends to overestimate the number of patients at high risk. Cardoso et al. found that Adjuvant! Online incorrectly classified 23% of patients as high clinical risk when Oncotype DX classified them as low genomic risk. [19].

In a population-based validation study, Olivotto et al. suggest that in patients under 35 years of age and who test positive for lymphovascular invasion, Adjuvant! Online would overestimate survival. It was also found that Adjuvant! Online tends to overestimate the survival rates of younger women with ER-positive breast cancer [3] and that it overestimated the added value of chemotherapy for older patients [20–22]. The validity of the predictive score calculated by Adjuvant! Online was deemed weak in the clinician-based validation [23]. Predictions on loco-regional relapse and distant metastases may vary greatly, making it difficult to make clear recommendations for adjuvant treatment [24]. This is reflected in two studies that suggest that when patients are involved in a discussion to decide on adjuvant chemotherapy, they are less likely to choose chemotherapy if using Adjuvant! Online [25, 26].

The ethnic variation in the data on which these online tools are based seriously affects the generalisability of these online tools. The SEER database is representative of the usual US population in terms of age, sex and ethnic distribution. However, the ethnic mix of the US population is different from that of England and Wales.

#### NHS PREDICT

The NHS PREDICT online tool is based on a cancer registry database of 5694 patients in the UK [4]. Unfortunately, independent investigators have raised concerns regarding the quality of the cancer registry data [27, 28]. Joishy et al. identified the lack of education of medical professionals and imprecision and inconsistency in medical records as factors negatively impacting the reliability of the data, and stated that insufficient time, personnel and finances had been allocated to ensure high quality [29]. The NHS PREDICT tool does not provide any estimate of local relapse and does not consider mortality due to causes other than breast cancer in its survival estimate. Therefore, a total reliance on the NHS PREDICT online tool may deprive patients, particularly those with small, biologically aggressive cancers, of the benefit of chemotherapy [24].

In our unpublished audit of 120 patients who underwent genomic profiling using both the EP Clin score and NHS PREDICT calculation, the disconcordance rate was significantly high (43%). If we relied solely on NHS PREDICT, a significant proportion of patients with small, node-negative tumours would not have received chemotherapy, despite needing adjuvant therapy according to the EP Clin score.

Wong et al. found that NHS PREDICT substantially overestimates survival in very young patients with breast cancer and those receiving chemotherapy [30].

As with Adjuvant! Online, the ethnic mix of the outcome data used to develop NHS PREDICT may not be generalisable to more diverse metropolitan areas in the UK, such as London and Birmingham.

In summary, online prediction tools continue to be of value as free adjuncts to therapeutic decision-making. However, the use of these tools should be tempered with recognition of the inherent biases of the underlying databases and the well-documented limitations of these algorithms, such as overestimation of the benefits of chemotherapy in certain patient groups, underestimation of the benefits of chemotherapy in patients with small, biologically aggressive tumours, lack of generalisability to more diverse populations, and lack of standardisation in clinical utility.

### Genomic assays

The use of genomic assays in human breast cancer has been endorsed by the National Institute for Clinical Excellence (NICE) and the American Society of Clinical Oncology (ASCO), among others [31]. Several histological and molecular markers are used to identify patients with breast cancer at the highest risk of recurrence, which also means tumours with the highest degree of sensitivity to chemotherapy [11].

The most established example of a genomic assay score is the recurrence score (RS), which is based on an Oncotype DX 21-gene assay panel. The RS ranges from 1 to 100, and is stratified into low risk (below 18), intermediate risk (18 to 30) and high risk (over 30). RS was the first such score to be included in NICE and ASCO guidelines [31, 32].

EndoPredict (EP) is a 15-point score based on an 8-gene panel which assigns patients to high- and low-risk groups (below and above 5, respectively). The reliability of the score is increased by incorporating clinical parameters (tumour size and nodal status) in a score that has been named EndoPredict Clinical (EpClin). EP and EpClin have been shown to provide more prognostic information than RS, in part due to the combination of genomic data with nodal status and tumour size. The absence of an intermediate risk group in EP makes decision-making more straightforward than with RS [33]. EpClin provides reliable information about the benefit of chemotherapy by combining molecular signatures with clinicopathological variables and data derived from randomised controlled trials [34].

Mammaprint is the oldest available test, consisting of a 70-gene assay that stratifies the patients into high- and low-risk groups [19]. A further 80-gene panel called Blueprint was developed for more accurate typing of breast cancer (5). It is meant to be used in conjunction with Mammaprint. Mammaprint is still waiting for external validation in the MINDACT trial, the results of which were presented at ASCO [35].

PAM50 is a 50-gene assay that identifies the breast cancer subtype, and generates a risk of recurrence score (ROR). The ROR is a 100-point scale that stratifies patients into low risk of recurrence, intermediate risk and high risk. PAM50 was validated in the ATAC and ABCSG-8 trials, where it was found to be superior to immunohistochemistry and RS in ER-positive node-negative breast cancer patients receiving endocrine therapy [8, 9].

The efficacy of genomic assays is a testament to the milestones achieved in the understanding of cancer biology and the recent recognition of the heterogeneity of breast cancer as a disease.

Internet-based mathematical and computational algorithms provide physicians and patients with useful information regarding prognosis and the benefits of systemic therapy. They are particularly valuable in resource-constrained international healthcare. However, their inherent limitations, which are related to the conceptual design, methodology and data quality, make these decision aids insufficiently robust to be used as an alternative to molecular profiling of the primary tumour in the modern era of personalised cancer care and precision medicine.

Early breast cancer is currently considered a systemic disease and a complex ecosystem with behaviour determined by complex genetic and molecular signatures of the tumour cells, mammary stem cells, microenvironment and host immune system rather than an anatomical neoplastic process that progresses locally and then spreads to regional lymph nodes and other organs during tumour progression. Therefore, we anticipate that molecular profiling will continue to evolve and expand to include the tumour microenvironment, cancer stem cells and host immune system, in addition to the primary tumour, to further refine therapeutic decisions and optimise clinical outcomes [36].

However, it would be prudent to closely follow developments in this field, as no single multi-gene assay is emerging as standard and no one technology is uniformly accepted. Continued studies for validation and reproducibility of genomic tests are needed to better understand their limitations and to further increase their utility in making treatment decisions in the early stages of breast cancer.
